# Hidden Hunger: Solutions for America’s Aging Populations

**DOI:** 10.3390/nu10091210

**Published:** 2018-09-01

**Authors:** Manfred Eggersdorfer, Ucheoma Akobundu, Regan L. Bailey, Julie Shlisky, Amy R. Beaudreault, Gilles Bergeron, Robert B. Blancato, Jeffrey B. Blumberg, Megan W. Bourassa, Filomena Gomes, Gordon Jensen, Mary Ann Johnson, Douglas Mackay, Keri Marshall, Simin Nikbin Meydani, Katherine L. Tucker

**Affiliations:** 1 DSM Nutritional Products AG, Human Nutrition and Health, 4002 Basel, Switzerland; 2 Department of Healthy Ageing, University Medical Center Groningen, 9713 GZ Groningen, The Netherlands; 3 Meals on Wheels, Arlington, VA 22202, USA; uche@mealsonwheelsamerica.org; 4 Department of Nutrition Science, Purdue University, West Lafayette, IN 47907, USA; reganbailey@purdue.edu; 5 The Sackler Institute for Nutrition Science, The New York Academy of Sciences, New York, NY 10007, USA; jshlisky@gmail.com (J.S.); gbergeron@nyas.org (G.B.); mbourassa@nyas.org (M.W.B.); fgomes@nyas.org (F.G.); 6 Department of Nutrition, UC Davis, Davis, CA 95616, USA; abeaudreault@ucdavis.edu; 7 National Coordinator, Defeat Malnutrition Today, Washington, DC 20006, USA; rblancato@matzblancato.com; 8 Jean Mayer USDA Human Nutrition Research Center on Aging and Friedman School of Nutrition Science and Policy, Tufts University, Boston, MA 02111, USA; jeffrey.blumberg@tufts.edu (J.B.B.); simin.meydani@tufts.edu (S.N.M.); 9 Larner College of Medicine, University of Vermont, Burlington, VT 05405, USA; gordon.jensen@uvm.edu; 10 Department of Nutrition and Health Sciences, University of Nebraska-Lincoln, Lincoln, NE 68583, USA; majohnson@unl.edu; 11 Council for Responsible Nutrition, Washington, DC 20036, USA; dmacKay@crnusa.org; 12 DSM Nutritional Products, Columbia, MD 21045, USA; keri.marshall@dsm.com; 13 Biomedical & Nutritional Sciences Department, University of Massachusetts Lowell, Lowell, MA 01854, USA; katherine_tucker@uml.edu

**Keywords:** nutrition, older adults, health, policy

## Abstract

The global population, including the United States, is experiencing a demographic shift with the proportion of older adults (aged ≥ 65 years) growing faster than any other age group. This demographic group is at higher risk for developing nutrition-related chronic conditions such as heart disease and diabetes as well as infections such as influenza and pneumonia. As a result, an emphasis on nutrition is instrumental for disease risk reduction. Unfortunately, inadequate nutrient status or deficiency, often termed hidden hunger, disproportionately affects older adults because of systematic healthcare, environmental, and biological challenges. This report summarizes the unique nutrition challenges facing the aging population and identifies strategies, interventions, and policies to address hidden hunger among the older adults, discussed at the scientific symposium “Hidden Hunger: Solutions for America’s Aging Population”, on March 23, 2018.

## 1. Introduction

Hidden hunger is commonly used to describe individuals who may have adequate energy consumption, but suboptimal micronutrient intakes, placing them at risk for nutrition-related diseases. When prolonged, inadequate dietary intake of micronutrients (vitamins and minerals) and macronutrients (fat, protein, and carbohydrates) can have adverse effects on health outcomes that may result in a cycle of sub-optimal health. Hidden hunger in the context of the United States may occur in those with normal weight as well as overweight or obese individuals. As hidden hunger may often not show physical symptoms, the condition can be easily overlooked by clinicians and is not always well diagnosed or documented. Malnutrition may be found at any point during the lifecycle, but older adult populations often receive inadequate attention regarding the existence or degree of hidden hunger.

The global population is amid a dramatic demographic shift as life expectancy at birth is continuing to increase and so is the proportion of older adults (≥65 years old). Indeed, older adults are the segment of the American population that is growing the fastest. In the United States, the oldest baby boomers (born 1946–1964) reached age 65 in 2010, and this age group is expected to grow 3.0% each year through the next two decades [1]. By 2050, this segment is projected to be 20.0% of the population, resulting in approximately 10,000 individuals turning 65 each day [2].

As older adults are at increased risk for infectious and chronic diseases [3], emphasis should be placed on primary and secondary prevention strategies to reduce risk of age-associated disease, including the key role of nutrition [4,5]. Improving healthcare policies and clinical education in this regard will be vital to increasing the quality of life of this growing population.

Determinants of healthy aging include individual and collective variables [6]. Individual cognitive and physiological alterations that manifest as functional impairments include aspects such as decreased taste and smell [7], loss of appetite [8], dental and chewing problems [9], and the physical ability to independently shop, cook, and eat. These changes may be influenced by the normal aging process, certain disease states, polypharmacy, surgical interventions and/or repeated hospitalization, and energy balance. Environmental and social factors include aspects such as social support and engagement, economic constraints, environmental exposure, and the built environment, which all have significant roles in mediating access to nutritious foods and to their adequate intake [10].

A leading cause of nutrition risk among older adults is food insecurity, which exists when people do not have reliable access to safe and nutritious food to lead an active, healthy life [11]. A 2015 study found that 9.8 million (14.7%) U.S. older adults faced the threat of hunger [12] and, according to a 2015 AARP (formerly American Association of Retired Persons) report, 10 states had food insecurity with a prevalence above 20% [13]. Food insecure older adults have been estimated to be 50% more likely to have diabetes, three times more likely to suffer depression, 60% more likely to have congestive heart failure or a heart attack, 30% more likely to have impairment of activities of daily living, and two times more likely to report gum disease and asthma [14].

In addition to the health consequences, the economic burden of sub-optimal nutrition among older adults is extremely high and growing, with the annual U.S. economic cost of malnutrition-associated diseases estimated at USD $51.3 billion for this population group [15]. Among hospital patients, there has been an approximate 300% increase in healthcare costs associated with patients with poor nutrition [16]. Much of the increased costs associated with malnutrition were driven by longer hospital stays and greater rates of complications, infection, readmissions, and falls [17]. Older adult patients already experience four to six extra days in the hospital compared to younger adult patients [5]. This could be further exacerbated when they are malnourished.

To understand the status of inadequate nutrition among the U.S. older adult population—and to review key challenges and program and policy solutions to encourage quality healthcare practices—The Sackler Institute for Nutrition Science, a program of the New York Academy of Sciences, convened a scientific symposium “Hidden Hunger: Solutions for America’s Aging Population” on March 23, 2018. The symposium involved expert presentations from speakers who play active roles in research, policy, and program implementation and evaluation.

This manuscript summarizes the symposium’s presentations, discussions, and recommendations, and aims to review the unique dietary needs and challenges of older adults and to identify strategies and interventions to address hidden hunger. The paper also includes additional considerations and clarifications from the symposium, drawing from the panel discussions and manuscript authors.

## 2. Unique Dietary Needs and Challenges Meeting the Needs of the Aging Population

The longevity of an individual is based on intrinsic (e.g., genetic), and extrinsic (e.g., lifestyle, diet, and environmental) factors. A significant change that occurs among older adults is physiological dysregulation, especially related to the immune system. The aging process can lead to increased inflammation, impaired innate immune response, and decreased T cell-mediated function. A dysregulated inflammatory response is associated with diseases such as Alzheimer, arthritis, type 2 diabetes, cancer, and other neurological, cardiovascular, pulmonary, and bone diseases. Additionally, a decrease in cell-mediated immunity predisposes older adults to infection, autoimmune diseases, arthritis, and cancer.

Poor nutrition is a major risk factor for the development of infectious and chronic diseases and suboptimal cognitive function [18], which can negatively impact a person’s quality of life and longevity [19]. However, emphasizing the consumption of certain foods, nutrients and dietary patterns that can promote healthy aging may reduce the risk or blunt the progression of a wide range of chronic diseases, and have a positive effect on health span.

### 2.1. Food, Nutrients, and Dietary Patterns for Healthy Aging

According to the U.S. Dietary Guidelines 2015–2020, important shortfall nutrients among older adult Americans include vitamin D, calcium, potassium, and dietary fiber [20]. The aging population is at risk for many additional nutrient inadequacies (Table 1), including sub-optimal intake of omega-3 fatty acids, folate, vitamins B_6_, B_12_, C, D, E, and K, magnesium, and zinc, all of which can be associated with health problems among the elderly. Protein is also uniquely important for older adults for preserving muscle mass with aging.

#### 2.1.1. Protein

As muscle mass naturally decreases with age and contributes to the higher risk for falls in older people, maintaining lean mass (LM) is imperative. The Health, Aging, and Body Composition study [22] found that community-dwelling men and women (aged 70–79) in the highest quintile of protein intake lost 40% less LM over time than those in the lowest quintile, concluding that dietary protein may be a modifiable risk factor for sarcopenia. The current Institute of Medicine (IOM) recommendations for protein intake remain the same throughout adulthood. However, it has been suggested that older adults may need moderately higher protein intake of 1.0–1.3 g/kg/day [23] to maintain nitrogen balance and offset the decreased efficiency of protein synthesis and impaired insulin action that come with aging and prevent muscle mass loss.

#### 2.1.2. Omega-3 Fatty Acids (Omega-3)

Adequate omega-3 intake is associated with decreased risk of heart disease, cognitive decline, and asthma. Optimal intake in food is a challenge, as the only major source of the omega-3 eicosapentaenoic acid (EPA) and docosahexaenoic acid (DHA) is fatty fish like salmon, mackerel, and sardines. Plant food sources of omega-3, alpha-linolenic acid (ALA), exist but are primarily limited to flax seeds and walnuts, and its transformation into the active EPA and DHA is inefficient in humans. Thirty-six percent of men ≥71 years and 18.0% of women do not meet the recommended intake of DHA, EPA, and ALA [21]. In the Framingham Heart Study [24], individuals with the highest levels of DHA in the blood had significantly lower cumulative incidence of dementia over a 12-year follow-up. Additionally, a study in Singapore found that higher intakes of EPA, DHA, and ALA were associated with reduced risk of cardiovascular mortality [25].

#### 2.1.3. Dietary Fiber

Dietary fiber plays an important role in gastrointestinal function. An unhealthy gut microbiota has been associated with infectious and degenerative diseases [26], specifically inflammatory bowel disease, autoimmune arthritis, obesity, and metabolic syndrome [27]. Fiber has been identified as a “nutrient of public health concern” as its intake in U.S. diets is very low, particularly among the lower income groups including non-Hispanic black men and non-Hispanic black, Hispanic, and Mexican American women [28]. At least 90% of older American’s (≥71 years old) are not consuming the recommended amounts daily due in part to highly refined diets.

#### 2.1.4. B Vitamins: Folate, B_12_, and B_6_

B vitamins are essential for many biological processes including neurological and immune function and optimal bone health. Folate is necessary for DNA methylation and prevents hyperhomocysteinemia, which is associated with inflammation, endothelial dysfunction, and cardiovascular disease. In the National Health and Nutrition Examination Survey (NHANES 2011–2012 [21]) 26% of women ≥71 years old had low intake of folate. Vitamin B_12_ absorption becomes less efficient with aging—leading to an increased risk of deficiencies—which may have severe consequences because of the vitamin’s role in cell generation and cognitive function. Vitamin B_12_ intake is low if animal protein intake is low; in addition, its absorption is affected by inadequacy of stomach acid, so deficiency may also develop with prolonged use of acid blocking medications. The Framingham Offspring Study [29] found 20% of non-supplement users and 8.2% of supplement users were deficient in vitamin B_12_ based on plasma measures. Another B vitamin, vitamin B_6_, is also associated with homocysteine concentration and immune function, playing an important role in cognition and depression. Vitamin B_6_ is important for protein metabolism, is easily lost in food processing, and not generally added back to the food supply (e.g., through food enrichment), leading to significant rates of inadequate intakes and status.

#### 2.1.5. Vitamin D

Vitamin D is essential for the efficient absorption of calcium to support mineralization of bone and in prevention of muscle weakness. It is an especially important micronutrient for reducing risk of osteoporosis and fractures from falls. As vitamin D can be synthesized through ultraviolet (UV) exposure and is limited in food sources, elderly individuals are at higher risk of deficiency in this vitamin, as they tend to spend more time indoors. Additionally, older people experience decreased skin synthesis and decreased capacity of the kidneys to convert the vitamin into its active form, further increasing the risk of vitamin D deficiency in this population. NHANES data show that more than 95% of older men and women do not receive the recommended intake of vitamin D from diet alone [21].

#### 2.1.6. Dietary Patterns

Overall, a balanced diet of whole foods that limits intake of refined grains, processed meat, sugar, solid fats, and sodium is ideal in reducing the risk of chronic diseases [30]. Table 2 provides a summary of key nutrients for healthy aging, including the nutrient function, food sources, and either risks associated with deficiency and/or the benefits of adequate intake. Optimal health requires dietary patterns that maintain a proper balance of nutrients, and the Dietary Approaches to Stop Hypertension (DASH) and Mediterranean diets are often cited as representing healthy dietary patterns. The DASH diet, which emphasizes fruits, vegetables, and low-fat dairy was found to be effective in reducing hypertension, [31,32], whereas the Mediterranean Diet is similar, but places more emphasis on sources of monounsaturated fatty acids (olive oil) and omega-3 fatty acids. In the United States, the Healthy Eating Index (HEI) is a measure of diet quality based on the recommendations in the Dietary Guidelines for Americans. The DASH, Mediterranean, and HEI dietary scores were compared in a cohort of older, low-income Puerto Rican adults in Massachusetts, U.S. The results demonstrated that the Mediterranean diet was most significantly associated with lower cardio-metabolic risk, waist circumference, body mass index (BMI), insulin resistance, and inflammation [33]. The same study also showed the Mediterranean Diet to have a protective role on cognitive function [34]. Additionally, anti-inflammatory diets—with lower intake of omega-6 fatty acids and higher intake of omega-3 fatty acids and antioxidants—have been shown to have beneficial effects in the treatment of inflammatory age-related chronic diseases, such as diabetes, obesity, and metabolic syndrome. The balance of macronutrients is an important consideration in anti-inflammatory diets, e.g., the balance of protein to glycemic load in a meal can alter the secretion of insulin and glucagon and the omega-6/omega-3 fatty acids ratio in the diet can alter the expression of inflammatory genes. Other nutrients, such as polyphenols, have anti-inflammatory actions and can be obtained from a diet rich in colorful, non-starchy vegetables [35].

### 2.2. Defining Hidden Hunger in the United States in the Context of Aging and Obesity

Importantly, hidden hunger can occur not only in the underweight, but also in the overweight and obese. A study of NHANES data (R.L. Bailey, personal communication) showed that 33.0% of men 65 years old or older were obese and 42.0% were overweight. Among women the same age, 36.0% were obese and 32.0% were overweight. Obese women had the highest percentage of food insecurity across all aging adults.

Obese adults and children have been shown to have lower micronutrient status than normal weight individuals. Kimmons et al. showed that prevalence of micronutrient deficiency, including vitamins C and E, carotenoids, folate, and B12 increased with increase in body mass index (BMI) [58]. Obese women had lower levels of folate, vitamins C, E, and D, as well as higher oxidative stress and inflammation compared to normal weight women [59]. Obese children were shown to have lower serum vitamin E and β-carotene levels [60]. In a study of elderly Ecuadorians, obesity was prevalent, and the obese elderly exhibited micronutrient deficiencies along with metabolic syndrome [61]. Unfortunately, adequate information related to micronutrient status of obese older adults in the United States is not available.

Aside from the typical outcomes associated with excess weight, such as type 2 diabetes and cardiovascular disease, obesity predisposes individuals to additional health concerns and diminishes health-related quality of life [62]. For example, obese men (10.1%) and women (7.1%) are more likely to have poor oral health, a primary challenge to a healthy diet [63]. In a 2004 Pennsylvania cohort study, chewing, swallowing, and mouth pain were associated with lower HEI, micronutrient risk, polypharmacy (≥ five medications), and chronic disease risk [9]. Although bone health was better in obese older adults, women were more likely to be deficient in vitamin D and vitamin B_12_ and to experience depression. Furthermore, obese older persons frequently suffer from destructive joint disease of the knees necessitating knee replacement surgery [64] and are at increased risk of infectious diseases.

## 3. Strategies and Interventions to Diagnose and Address Hidden Hunger

Many seniors are not able to leave their homes and are considered homebound because of functional disabilities, multiple comorbidities, depression, and/or cognitive impairment. These conditions create unique challenges in meeting the dietary needs of older adults, especially as they contribute to increased risk for social, physical, and emotional isolation. A 2011 study conducted in the U.S. found that approximately two million Medicare recipients were completely or mostly homebound, and these individuals were more likely to be older than the non-homebound individuals [65]. Targeting appropriate in-home medical and preventative services to this population would be beneficial in improving their health and quality of life [62], possibly delaying premature institutionalization and utilization of high-cost healthcare [66].

In addition to being homebound, older adults are at increased risk of falling. Approximately 30% of older adults experience a fall each year, resulting in bone fractures, resulting in an estimated economic cost of approximately USD $50.0 billion [67]. However, the cost of delivering one nutritionally-dense meal per day/person for a year is approximately the same cost as one day in a hospital/person [68,69] and may help prevent the nutritional deficiencies that predispose older adults to falling [70,71]. Home-delivered meal programs have been proven to improve dietary quality and nutrient intake [72]. Senior nutrition programs across the country, including Meals on Wheels America, help address many of the health challenges faced by seniors by providing coordinated care and reducing social isolation, while supporting nutritional quality, medication adherence, fall prevention, chronic health management, transportation, and caregiver support. In addition to Meals on Wheels America, the U.S. Federal government and the AARP Foundation support nutrition for older adults through various programs (Table 3 provides some examples). Evaluating the effectiveness of these nutritional programs similar to evaluations conducted by the Administration for Community Living and Meals on Wheels America, will provide much needed information in support of adequate nutrition to expand the health-span of older adults [73].

In addition to nutrition programs that are available at the population level, targeted and proactive nutrition screening can help identify older adults who are at nutrition risk. Dietary assessment is not practical in a clinical setting, but a number of screening tools have been developed to examine nutrition risk for inpatient and outpatient settings. The Dietary Screening Tool is a validated screener that has been related to food group and nutrient intakes as well as biomarkers of nutritional status [74]. These screening methods, however, are insufficiently sensitive to identify compromised micronutrient status in independently living older adults without overt signs of nutrient deficiencies. Assessing micronutrient status as part of the regular physical exam of older adults will help identify those at risk for nutrient deficiencies and provide opportunity for targeted intervention cost effective intervention.

### 3.1. Effect of Dietary Supplementation and Polypharmacy

The Dietary Guidelines for Americans 2015–2020 [20] categorized folate, calcium, magnesium, potassium, dietary fiber, and vitamins A, C, D, and E as “shortfall nutrients” (i.e., under-consumed). Even with a diverse diet, nutritional gaps can exist but they can be mitigated through food enrichment (replacement of nutrients lost in processing), fortification (adding nutrients), and/or use of dietary supplements. Although limited information is available in the United States on nutrient source intake, it is estimated that in 2011–2012 52% of Americans used a dietary supplement and 31% took a multivitamin/multimineral supplement (MVMS) [83]. In older adults, supplement intake increased with age (64.9% age 51–70 years and 73.2% age ≥71 years) [84]. Among the supplement users, 29% took four or more products [85].

While the debate continues regarding the efficacy of dietary supplement use, MVMS use was associated with fewer micronutrient inadequacies, especially in older adults [86,87]. Adult consumers (ages 51–70 years) of supplements had higher intakes of vitamins A, C, and E and lower intake of choline, whereas consumers ≥71 years old had higher intakes of copper, magnesium, vitamins B_6_, and K when compared to individuals who did not take supplements [84]. Unfortunately, those who are most at nutrition risk are those who are less likely to use dietary supplements [88].

Polypharmacy can also affect absorption or metabolism of micronutrients. This is an increasingly important issue with older adults as 39.7% of those ≥65 years took five or more prescription drugs in the previous 30 days [89]. The most common prescription drugs used among those ≥65 years old are antidiabetics, anti-acid reflux, and cardiovascular medications (including acetyl choline esterase (ACE) inhibitors, beta-blockers, calcium channel blockers, and diuretics) [89]. Chronic use of antacid medications decreases absorption of folate, vitamin B_12_, iron, magnesium, and zinc, whereas type 2 diabetes medication can lower folate and vitamin B_12_. Diuretics, commonly prescribed for cardiovascular diseases, can decrease absorption of calcium, potassium, magnesium, zinc, B_6_, and thiamine.

Nutrient-containing dietary supplements are advantageous as part of the solution to tackle malnutrition in the older population because they are a relatively rapid, economic, and specific intervention (particularly if combined with micronutrient assessment) to alleviate gaps in under-consumed nutrients. Importantly, MVMS do not increase caloric intake, nor do they require changes in the food supply to be effective in improving nutritional status. The need for education about dietary supplements and subsequent behavior change is modest compared to other interventions to promote health and reduce the risk of chronic diseases. Limitations for supplement use include the added cost to the consumer, the potential for poor adherence, and an inability to reach some at-risk individuals. Furthermore, whereas MVMS are helpful in filling micronutrient gaps and improving nutritional status, it is important to note that they do not contain dietary fiber or the myriad of phytonutrients important for health. As with all such products, MVMS are meant to supplement and not act as substitutes for a good quality diet.

## 4. Bringing Attention to Policymakers and Public Health Experts

The roles of research and advocacy organizations are critical to maintaining and expanding policies focused on improving nutrition for older adults. To implement meaningful changes for our nation’s seniors, it is essential to bring the major nutrition-related issues faced by this population to key policymakers, many of whom may not be aware of these issues in their constituency. When approaching policymakers, providing the necessary background materials to aid in education is essential. An important aspect in education is to have evidence-based research (inclusive of information regarding cost savings or return on investment) to support the initiative in non-scientific terms and to share how the initiative will improve the lives and health of the policymakers’ constituents. After an introduction, it is important to further cultivate policymakers through continued communication. Throughout the process, maintain progress toward the objective by scheduling a formal meeting, hearing request, bill introduction, and/or regulatory initiative. By using a consistent and simple message, decision-makers are more likely to become familiar with the need for the proposed action.

To advocate for an issue, such as ending hidden hunger in the older adult population, it is important to remain adaptable to changes that may be necessary to keep progress moving forward and to always understand the environment and the appropriateness of the request. Be familiar with relevant terms and preemptive with possible questions, such as value and return on investment. In addition, be familiar with the backgrounds of legislative or executive branch champions of the issue.

Lastly, action is necessary. Work to build a broad stakeholder base at the start with various partners with common goals to increase the impact of the message. In today's environment, a two-sided issue unfolds: proactive progressive advocacy for innovative policies, or defensive advocacy to preserve and protect what is currently in place.

## 5. Discussion and Recommendations

Consuming a diverse nutrient-dense diet is imperative throughout the aging process to maintain and promote health. The nutrient quality of food is essential because poor nutrition is a leading cause of many infectious and chronic diseases in older adults. Due to the demographic shift toward an older population globally, attention to optimal dietary patterns and accurate assessment of nutrient status among the older adults should become a priority in clinical settings and policy development.

Many challenges exist in identifying and preventing malnutrition and inadequate nutrition among older adults. Firstly, a collective understanding of the distinct nutritional needs of older adults is lacking, and federal funding has been stagnant or inadequate for fully understanding this complex issue. Exploring various funding sources outside federal funds should be explored to further research in this area. Furthermore, policymakers should consider the inherent challenges and costs of nutritional screening and identifying and targeting services to those in need as compared to the significantly greater costs and consequences of hidden hunger in older adults.

Despite these challenges, an improved scientific understanding of the role of good nutrition in infectious and chronic disease risk reduction and self-management is creating a significant opportunity. By leveraging the strengths of various stakeholder networks, new products and delivery models are being created as potential solutions. Technological innovations assist in education support, engagement, empowerment, and food access facilitation. In addition, the increased awareness of the role of social determinants on health among healthcare entities is enabling a more systems-based approach to meet the unique needs of the older adult population.

Additional research is necessary, as well as collecting and leveraging available evidence to inform advocacy, policy, and community action. Research priorities for reducing hidden hunger in older adults include: (1) identifying threats to the nutrition status of community residing older adults; 2) documenting the extent and prevalence of specific nutrient deficiencies among a heterogeneous population of older adults; (3) identifying and quantifying the risks and benefits of potential interventions; (4) establishing the impact, cost, consequence, and opportunity offered by various interventions; and (5) using this information to inform public policy and ongoing research activities. Due to the improvement and prioritization of interdisciplinary and cross-sectorial efforts in nutrition and public health, unique partnerships have the capacity to develop innovative research and evidence-based policy to target and improve the health of this growing population. This symposium was an active step in bringing together stakeholders from various backgrounds to draw attention to this at-need population for improved nutrition interventions at-home and in clinical settings.

## Figures and Tables

**Table 1:** Proportion of adults ≥71 years old with low nutrient intakes (NHANES 2011–2012) [21].

Adults ≥71 Years Old Who do not Meet the Daily Recommended Intake (% < EAR or AI *)				
	Men (*n* = 327)		Women (*n* = 322)	
Nutrient	Cutoff point	% (SE)	Cutoff point	% (SE)
Dietary fiber *	30 g	92 (3)	21 g	90 (4)
Omega-3 Fatty Acids *	1.6 g	43 (9)	1.1 g	21 (8)
Folate (DFE)	320 μg	4 (2)	320 μg	26 (5)
Vitamin B6	1.4 mg	7 (3)	1.3 mg	38 (6)
Vitamin B12	2.0 μg	0	2.0 μg	9 (4)
Vitamin C	75 mg	53 (9)	60 mg	43 (7)
Vitamin D	10 μg	95 (4)	10 μg	97 (2)
Vitamin E	12 μg	74 (6)	12 μg	97 (1)
Vitamin K *	120 μg	71 (6)	90 μg	53 (7)
Calcium	1000 mg	65 (6)	1000	78 (5)
Magnesium	350 mg	65 (7)	265 mg	64 (5)
Potassium *	4700 mg	99 (1)	4700 mg	100
Zinc	9.4 mg	16 (5)	6.8 mg	30 (7)

Estimated using the National Cancer Institute Method with NHANES 2011–2012 data; based on food intake only. EAR: estimated average requirement, AI (*): adequate intake, DFE: dietary folate equivalents.

**Table 2:** Evidence for optimal nutrition in disease and infection prevention in older adults.

Nutrient	Function	Recommended Sources	Associated Risks/Benefits
Protein	Muscle mass	Low-fat dairy, fish legumes, nuts, lean meat/poultry	Deficiency associated with loss of muscle mass, decreased immunity, and weakening of the heart and respiratory system [36]
Omega-3 Fatty Acids (EPA/DHA/ALA)	Chronic disease and cognitive decline prevention	Walnuts, fatty fish, flaxseeds	Associated with reduced risk of cardiovascular mortality [25], inflammation ,and prevention of Alzheimer's Disease (observational longitudinal data) [37]
Dietary fiber	Intestinal and metabolic health	Whole grains, fruits, and vegetables	Lowers cholesterol and improves glycemia, weight loss, and stool normalization [38] Alters microbiota composition, lowering chronic inflammation, and improving gut barrier function [39]
Folate	Cell division	Whole grains, fruits, and vegetables	Associated with decreased risk of colorectal cancer [40]
Vitamin B_6_	Metabolism	Fish, legumes, nuts, whole grains, fruits, and vegetables	Deficiency associated with microcytic anemia, dermatitis with cheilosis and glossitis, depression, confusion, and weakened immune function [41]
Vitamin B_12_	Nerve and blood health	Low-fat dairy, shellfish and fish, fortified cereals, lean meat/poultry	Deficiency associated with poor cognition, anemia, and hyperhomocysteinemia [42] Deficiency leads to peripheral neuropathy, balance disturbances, cognitive disturbances, and disability [41]
Vitamin C	Biosynthesis of collagen, L-carnitine, and certain neurotransmitters; protein metabolism	Fruits and vegetables	Reduces risk of cataract [43] Deficiency impairs collagen synthesis and wound healing [44]
Vitamin D	Bone, neurological health	Low-fat dairy (fortified)	Vitamin D with calcium reduces risk of total factures (15%) and hip fracture (30%) [45] Deficiency associated with osteoporosis, neurologic conditions, diabetes, and other metabolic conditions
Vitamin E	Immune function maintenance	Nuts, wheat	Deficiency associated with reduced risk of age-related cataract [46] Reduces risk of acquiring respiratory infections [47] Prevents or delays coronary heart disease
Vitamin K	Blood clotting, bone health	Green leafy vegetables, soybeans, nuts	Deficiency associated with reduced bone mineralization potentially contributing for osteoporosis [48], and development of cardiovascular disease [49]
Calcium	Skeletal health, blood pressure control	Low-fat dairy	Impact of dietary protein on the skeleton is most favorable in those who meet dietary calcium requirements [50] Increases overall bone mineral density and femoral neck bone mineral density [51]
Iron	Oxygen transport	Whole grains, pulses, nuts, lean meat, seafood, vegetables	Most common cause of anemia; associated with cancer, heart failure, gastrointestinal, and liver disorders [52]
Magnesium	Bone health, blood pressure, regulates calcium and potassium	Low-fat dairy, legumes, fruits, and vegetables	Deficiency associated with increased risk of prediabetes and diabetes [53] With age, gut absorption decreases and renal excretion increases
Potassium	Cellular function	Low-fat dairy, fruits, and vegetables	Insufficient intake contributes to hypertension, cardiovascular disease, kidney stones, and osteoporosis [54]
Zinc	Cellular metabolism	Whole grains, beans/pulses, lean meat/poultry, nuts	Decreases incidence and duration of pneumonia based on the number of new antibiotic prescriptions and days of antibiotic use [55] Decreases incidence of infections [56] Accelerates wound healing [57]

EPA: eicosapentaenoic acid; DHA: docosahexaenoic acid; ALA: alpha-linolenic acid.

**Table 3:** U.S. food-related programs and associations for older adults.

Program	Purpose	Outcomes
Supplemental Nutrition Assistance Program (SNAP)	Provides eligible, low-income individuals/families a monthly supplement for purchasing nutritious food (through a debit card to use for groceries).	5.1 million people ≥60 years old (11.8% of recipients) [75]
		Less likely than similar non-participants to forego their full prescribed dosage of medicine because of cost [76]
		Enables low-income seniors to live independently in their communities and avoid hospitalization [76]
Older Americans Act Nutrition Program (OAANP)	Federal program that provides grants to states to help support nutrition services for older people throughout the country	142.5 million home delivered meals [77]
		79.45 million congregate meals [77]
		2.43 million people served [77]
		1.47 million received health promotion services [77]
		35,578 people received nutrition counseling [77]
Senior Farmers’ Market Nutrition Program (SFMNP)	Federal grant program to states used to give low-income seniors coupons that can be used to purchase fresh fruits, vegetables, herbs, and honey from farmers’ markets, roadside stands, and community supported agriculture programs	Provided over USD $20.58 million in assistance to elderly Americans (2014) [78]
		816,000 older Americans in 42 states, the District of Columbia, Puerto Rico, and 8 Indian Tribal Organizations received annual SFMNP voucher worth an average of USD $32 [79]
Commodity Supplemental Food Program (CSFP)	Federal program administered by state agencies to improve the health of low-income elderly persons ≥60 years by supplementing their diets with nutritious USDA Foods	Approximately, 630,000 participated each month from 48 states, District of Columbia, Puerto Rico, and 2 Indian Tribal Organizations [79]
The Emergency Food Assistance Program (TEFAP)	Federal program that helps supplement the diets of low-income Americans, including elderly people, by providing them with emergency food and nutrition assistance at no cost	Total food entitlement fund USD $29,175,695 (2017) [80]
Meals on Wheels America	Leadership nonprofit supporting the more than 5000 community-based programs across America that are dedicated to addressing senior isolation and hunger	Meals on Wheels America member programs across the country provide a cost-effective solution that addresses the biggest threats to health and well-being of older adults. Malnutrition, social isolation and in-home safety hazards not only jeopardize seniors, but place a significant financial strain on our country’s healthcare system
AARP Foundation	Large nonprofit with “Drive to End Hunger” campaign that raises awareness about food insecurity among older adults, meeting the immediate daily food needs of hungry seniors, and working to establish permanent solutions to end senior hunger	Donated over 37 million meals to help hungry seniors across the country, since 2011 [81] The AARP Foundation’s Fre$h Savings and Grocery Guides programs provided over 12,000 seniors with added SNAP benefits or nutritional education in 2016 [81] Developed the “Screen and Intervene” online course to inform health care providers and community-based partners how to screen older patients for food insecurity and connect them to crucial resources [82]
